# Mussel-Inspired Anisotropic Nanocellulose and Silver Nanoparticle Composite with Improved Mechanical Properties, Electrical Conductivity and Antibacterial Activity

**DOI:** 10.3390/polym8030102

**Published:** 2016-03-22

**Authors:** Hoang-Linh Nguyen, Yun Kee Jo, Minkyu Cha, Yun Jeong Cha, Dong Ki Yoon, Naresh D. Sanandiya, Ekavianty Prajatelistia, Dongyeop X. Oh, Dong Soo Hwang

**Affiliations:** 1 Division of Integrative Bioscience and Biotechnology, Pohang University of Science and Technology, Pohang 790-784, Korea; linhpohang@postech.ac.kr (H.-L.N.); naresh@postech.ac.kr (N.D.S.); 2 Department of Chemical Engineering, Pohang University of Science and Technology, Pohang 790-784, Korea; yoonkee1988@postech.ac.kr; 3 Pohang Jecheol High School, Pohang 790-784, Korea; mkcha98@gmail.com; 4 Graduate School of Nanoscience and Technology and KAIST Institute for the NanoCentury (KINC), Korea Advanced Institute of Science and Technology (KAIST), Daejeon 305-701, Korea; yjc0925@kaist.ac.kr (Y.J.C.); nandk@kaist.ac.kr (D.K.Y.); 5 School of Interdisciplinary Bioscience and Bioengineering, Pohang University of Science and Technology, Pohang 790-784, Korea; ekavianty@postech.ac.kr; 6 Research Center for Industrial Chemical Biotechnology, Korea Research Institute of Chemical Technology (KRICT), Ulsan 44429, Korea; 7 School of Environmental Science and Engineering, Pohang University of Science and Technology, Pohang 790-784, Korea

**Keywords:** cellulose nanofibers, silver nanoparticles, anisotropic alignment, mechanical properties, electrical conductivities, antibacterial activities

## Abstract

Materials for wearable devices, tissue engineering and bio-sensing applications require both antibacterial activity to prevent bacterial infection and biofilm formation, and electrical conductivity to electric signals inside and outside of the human body. Recently, cellulose nanofibers have been utilized for various applications but cellulose itself has neither antibacterial activity nor conductivity. Here, an antibacterial and electrically conductive composite was formed by generating catechol mediated silver nanoparticles (AgNPs) on the surface of cellulose nanofibers. The chemically immobilized catechol moiety on the nanofibrous cellulose network reduced Ag^+^ to form AgNPs on the cellulose nanofiber. The AgNPs cellulose composite showed excellent antibacterial efficacy against both Gram-positive and Gram-negative bacteria. In addition, the catechol conjugation and the addition of AgNP induced anisotropic self-alignment of the cellulose nanofibers which enhances electrical and mechanical properties of the composite. Therefore, the composite containing AgNPs and anisotropic aligned the cellulose nanofiber may be useful for biomedical applications.

## 1. Introduction

There are increasing interests in biomedical electronics such as electronic skins, wearable healthcare sensors, and wearable human-device interfaces. The base materials for the next-generation wearable electronic systems should possess biocompatibility, antibacterial activity, surface electrical conductivity, and appropriate flexibility and toughness.

Recently, with the aforementioned concerns of the base materials for human healthcare device, cellulose has emerged as a base material because it is cheap, abundant, and has no cytotoxicity. In particular, a single thread of nanofibrous cellulose not only exhibits great mechanical property of ~120 (GPa) [1,2], it is also light in weight [3] compared to Kevlar or steel [4]. However, at a nanoscale point of view, cellulose disintegration is an energy-consuming process, due to intra- and interchain hydrogen bonds between the cellulose chains [5,6]. Carboxylation of cellulose fibrils by (2,2,6,6-tetramethyl-piperidin-1-yl)oxyl (TEMPO), a catalyst for selective oxidization, is a facile chemical pretreatment method for physical disintegration of cellulose fibrils [7]. Individual carboxylated cellulose nanofibers (CCNF) generated by TEMPO-mediated carboxylation have a high aspect ratio with an average width of about 3–5 nm and length of a few µm [6]. CCNF could generate transparent and flexible films with abundant carboxyl and polar hydroxyl functional groups, rigid and self-standing hydrogels, and aerogels with high surface area [6]. However, poor mechanical properties due to weak inter-fibril interaction, low bacterial resistance, and low electrical conductivity have impeded the application of CCNF to biomedical or wearable devices. Thus, many studies have focused on chemical modification to enhance inter-fibrillar interaction [8], biological activities [9] and electrical conductivity [10] of CCNF.

Dopamine (DA), a mussel-inspired building block that contains the key adhesive chemistry of l-3,4-dihydroxyphenylalanine (l-DOPA) and lysine, is known to form a material-independent adhesive coating via oxidative self-polymerization under wet conditions [11]. The catechol moiety of DA can also induce mineralization of metal nanoparticles as a green reducing agent [12,13,14,15]. DA can be easily conjugated onto a CCNF surface through amidaition reaction using 1-ethyl-3-(3-dimethylaminopropyl) carbodiimide (EDC) and *N*-hydroxysuccinimide (NHS) coupling catalysts over the carboxyl groups of CCNF. The catechol moiety of DA can lead to the reinforcement of binding between CCNFs by reducing and overcoming electrostatic repulsive forces. In addition, the catechol moiety can reduce Ag^+^ and form silver nanoparticles (AgNPs) on the cellulose nanofiber without additional chemical treatments or heating. AgNPs have been incorporated into various cellulose-based materials such as bacterial cellulose, filter paper, cotton fabric, and cellulose gels to create remarkable electrical conductivity and antibacterial activity [16,17,18]. However, additional chemical treatments and/or heating are generally required for the Ag^+^ reduction in the cellulose materials. In addition, 3-dimensional architecture of cellulose nanostructure due to AgNPs incorporation has been poorly studied.

**Figure 1:**
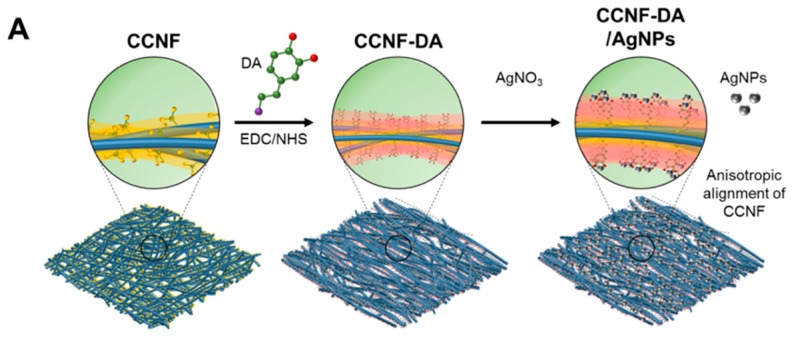

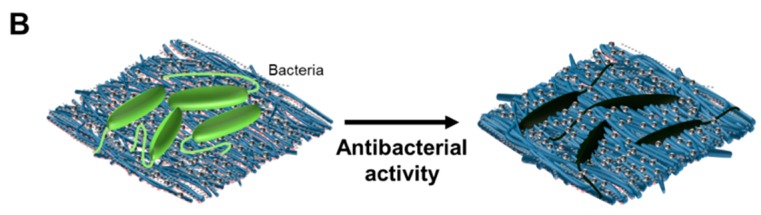
Schematic figures of (**A**) the anisotropic carboxylated cellulose nanofibers (CCNF)-dopamine (DA)/silver nanoparticles (AgNPs) composite formation process; and (**B**) the antibacterial activity of CCNF-DA/AgNPs composite.

In this study, we developed AgNP-containing cellulose nanofiber composites with improved mechanical property, antimicrobial activity, and electrical conductivity by exploiting mussel’s catechol chemistry. Interestingly, the composite of AgNPs and DA-conjugated CCNF (CCNF-DA) via *in situ* reduction of silver ions in silver nitrate (AgNO_3_) solution (Figure 1A) was anisotropically aligned due to the strong interaction between catechol moieties and AgNPs. The anisotropically aligned CCNF-DA/AgNPs composite can be applied to electrically conductive biomaterials with beneficial features including flexibility, electronic conductivity, and antibacterial activity (Figure 1B).

## 2. Materials and Methods

### 2.1. Materials

Commercial coffee filter papers (Kalita, Seongnam, Korea) from wood pulp were used to produce cellulose nanofibrils. TEMPO was purchased from Tokyo Chemical Industry (TCI, Tokyo, Japan). 1-Ethyl-3-(3-dimethylaminopropyl)carbodiimide hydrochloride (EDC·HCl), *N*-hydroxysuccinimide (NHS), sodium bromide (NaBr), AgNO_3_ solution, hydrochloride (HCl) solution, sodium hydroxide (NaOH), l-DOPA, dopamine hydrochloride (DA·HCl), and sodium nitrate (NaNO_3_) were purchased from Sigma-Aldrich (St. Louis, MO, USA) and were used as received.

### 2.2. Preparation of Carboxylated Cellulose Nanofibers (CCNF)

CCNFs were prepared by TEMPO mediated oxidation as described elsewhere [19]. In brief, cellulose from coffee filters (20 g) was mixed with TEMPO (0.312 g), and NaBr (2.058 g) in 2 L of distilled water (DW), and then NaClO (200 mmol) was added to the solution. The reaction was maintained at pH ~10 for several hours by adding 0.5 M NaOH or HCl. When the reaction is complete, the surface oxidized cellulose fibers were filtered and then washed 3 times with DW. The obtained slurry was dispersed in 2 L of DW and ground with a super masscolloider (MKCA6-2J; Masuko Sangyo Co., Ltd., Tokyo, Japan) at 15,000 rpm. The carboxyl group content (mmol·g^−1^, dry mass cellulose) of CCNF was analyzed through conductometric titration (Orion, Thermo Scientific, Waltham, MA, USA), and it was ~1.5 mmol·g^−1^. The weight concentration of CCNF (wt %) was measured with a moisture analyzer (MB 35, Ohaus, Parsippany, NJ, USA).

### 2.3. Preparation of CCNF-Dopamine (DA)

CCNF-DA was prepared following the previous study with minor modification [20]. In brief, EDC·HCl (1 equivalent) was directly added to the CCNF hydrogel, followed by addition of NHS (1 equivalent) and DA·HCl (2 equivalent) with stirring at 4 °C. The molar ratio of EDC·HCl, NHS, and DA·HCl was based on the carboxyl group molar content of CCNF. The mixture was stirred for 24 h and dialyzed with DW at pH 4–5 at room temperature. The suspension was stored in argon purged vials until further use. The catechol content of the CCNF-DA was quantified using a colorimetric assay developed by Arnow [21]. The CCNF-DA was added with 100 μL of DW, 300 μL of 0.5 M HCl, 300 μL of nitrite molybdate reagent (1.45 M sodium nitrite and 0.41 M sodium molybdate), and 300 μL of 1 M NaOH. The absorbance was measured at 500 nm using UV–Vis spectroscopy (PerkinElmer, Waltham, MA, USA) and the concentration of catechol was determined by Arnow’s standard curve of l-DOPA. To cast films, the CCNF-DA was poured into plastic petri dishes covered with adhesive Teflon tape and dried in an oven at 40 °C–50 °C for at least one day. The films were stored in a vacuum package prior to use.

### 2.4. Preparation of CCNF-DA/Silver Nanoparticles (AgNPs)

The solution of AgNO_3_ (3.25 mM) in acetate buffer (pH 4) was added to the CCNF-DA suspension. The mixture was sealed and left at room temperature for one day for reduction of Ag^+^ ions via oxidation of catechol groups. The film of CCNF-DA/AgNPs was fabricated following the method described above.

### 2.5. Characterization of CCNF-DA/AgNPs Film

CCNF, CCNF-DA, and CCNF-DA/AgNPs hydrogels with the concentration of 1 (*w*/*v*) % were analyzed by a cryogenic transmission electron microscopy (cryo-TEM; JEOL JEM 1011, Tokyo, Japan) and a polarized optical microscopy (POM; Nikon LV100 Pol) before the film casting. The samples were covered with the flat side of another B-type planchette and were rapidly frozen in a Bal-Tec HPM 010 high-pressure freezer (Boeckeler Instruments, Tucson, AZ, USA). After freeze substitution for 5 days at −80 °C in anhydrous acetone containing 2% OsO_4_, the samples were warmed up to room temperature over 2 days (24 h from −80 °C to −20 °C, 20 h from −20 °C to 4 °C, 4 h from 4 °C to 20 °C). After washing 3 times with anhydrous acetone, the samples were embedded in a graduated Epon resin (Ted Pella Inc., Redding, CA, USA) and diluted in acetone (5%, 15%, 25%, 50%, 75% and 100% (*v*/*v*)) over 3 days. After polymerization in a 60 °C oven for 24 h, the samples were sectioned and post-stained with aqueous 2% (*v*/*v*) uranyl acetate (UA) and Reynolds lead citrate (LC) solution. The resin was cut into 200 nm-sections using Leica EM UC7 (Wetzlar, Germany) and applied onto copper grids. The TEM images of samples were recorded. The optical textures were characterized by depolarized transmitted light microscopy (DTLM).

### 2.6. Characterization of AgNPs

For UV–Visible spectroscopy analysis, CCNF-DA/AgNPs films were immersed with ultra-pure water in a PCR tube, followed by ultrasonication to obtain the AgNPs from films. The absorbance of the soluble extract was measured using a UV–Visible spectrophotometer (OPTIZEN POP BIO, Mecasys, Daejeon, Korea) in a wavelength range of 350–600 nm. A few drops of NaNO_3_ were added to the extract to prevent aggregation of AgNPs [22].

For transmission electron microscopy (TEM) analysis, the CCNF-DA/AgNPs suspension was centrifuged at 9000 rpm for 10 min, followed by removal of the supernatant [23]. The aliquots of sample pellet (5–20 µL) were applied onto a copper microgrid, and the excess liquid was removed using filter paper. Subsequently, the sample was dried in a vacuum oven for 2 days. TEM images, selected area electron diffraction (SAED) pattern and energy-dispersive X-ray spectroscopy (EDS) data were obtained through high-resolution transmission electron microscopy (HRTEM; JEOL JEM-2100F, Tokyo, Japan). The results were further analyzed using image analysis software (ImageJ v.148; National Institutes of Health, Bethesda, MD, USA).

An inductively coupled plasma spectrometer (ICP; SHIMADZU ICPE-9000, Tokyo, Japan) was used to characterize the AgNPs content in the CCNF-DA/AgNPs.

The AgNPs release of the CCNF-DA/AgNPs composite in aqueous medium was investigated as follows. The composite film was incubated in phosphate-buffered saline (PBS) buffer for 2 days. The composite film was washed with deionized water and fully dried in a convection oven for 1 day. The AgNPs weight % in the incubated CCNF-DA/AgNPs composite film was measured using ICP.

### 2.7. Mechanical Properties and Conductivity Test

The CCNF-DA and CCNF-DA/AgNPs films were cut into circles with a width of ~5 mm and a thickness of ~40 µm. The mechanical properties of the composite films were measured on a universal tensile tester (UTS, Instron, Norwood, MA, USA). The composite films were loaded to failure at a strain rate of 0.01 s^−1^. Conductivities of the composite films were measured with a four-point probe. All measurements were replicated 5 times.

### 2.8. Antibacterial Activity Assay

The antibacterial activity of the CCNF-DA/AgNPs film was measured using Kirby-Bauer method for Gram-negative (*Escherichia coli*, ATCC 25922) and Gram-positive (*Staphylococcus aureus*, ATCC 6538) bacteria. The CCNF film was used as a negative control. The sample films were placed on Luria–Bertani (LB) agar plates containing bacterial cells in log-phase, and incubated overnight at 37 °C. The area of the inhibition zone was measured in triplicate using image analysis software (ImageJ v.148; National Institutes of Health, Bethesda, MD, USA).

To generate growth curves on the CCNF-DA/AgNPs, the sample films were incubated in LB medium inoculated with the bacterial cells in log-phase at a ratio of 1:100 (*v*/*v*). In addition, to determine a death curve, the films were incubated with 1 × 10^9^ cells·mL^−1^ of *E. coli* in 500 μL of LB medium in 24-well culture plate (SPL Life Science, Pocheon, Korea). For the growth/death curves, each bacterial strain was incubated at 37 °C and 300 rpm, and bacterial concentrations were monitored by measuring optical density at 600 nm (OD_600_) for 24 h.

### 2.9. Sustainability Test for Antibacterial Activity

The repeated growth-inhibiting and bactericidal activity of the CCNF-DA/AgNPs film was evaluated for up to 5 days, following a method described previously [24]. To determine growth-inhibiting efficacy, the CCNF-DA/AgNPs film was incubated overnight in 500 μL of LB medium inoculated with the *E. coli* cells in log-phase at a ratio of 1:100 (*v*/*v*). For bactericidal efficacy, the film was incubated overnight in 500 μL of LB medium containing 1 × 10^9^ cells·mL^−1^ of *E. coli*. The culture broth was sampled, and viable bacterial cells were counted at the end of each incubation period. Then, the film was gently washed with DW to remove remaining cells. The CCNF-DA/AgNPs film was incubated again as described above, and the process repeated for 5 days. The growth-inhibiting and bactericidal efficacies were calculated following (Equation 1) and (Equation 2).

Growth-inhibiting efficacy (%) = (*M* − *N*)/*M* × 100 (1)

Bactericidal efficacy (%) = (*N*_0_ − *N*)/*N*_0_ × 100 (2)

where, *N* is the mean number of surviving bacterial colonies in the sampled culture broth incubated on the CCNF-DA/AgNPs film; *M* is the mean number of colonies in the sampled culture broth incubated without the CCNF-DA/AgNPs film (control); and *N*_0_ is the initial mean number of colonies in the culture broth inoculated on the CCNF-DA/AgNPs film.

## 3. Results and Discussions

### 3.1. Anisotropic Structure of the CCNF-DA/AgNPs Film

CCNF-dopamine derivative (CCNF-DA) was synthesized by dopamine conjugation onto the carboxyl groups of CCNF via EDC/NHS chemistry [25]. The dopamine content in the CCNF-DA was 0.84 mmol·g^−1^ (dry cellulose weight) as determined by Arnow’s assay. This suggests that ~56 mol % of the carboxyl group on the CCNF were conjugated with DA. After preparing CCNF and CCNF-DA, three types of cellulose-based composite films were prepared using CCNF, CCNF-DA, CCNF-DA/AgNPs. The CCNF, CCNF-DA, CCNF-DA/AgNPs films were white, yellow, and gray, respectively. Both CCNF and CCNF-DA were transparent, but CCNF-DA/AgNPs was opaque due to the light absorption of AgNPs.

**Figure 2:**
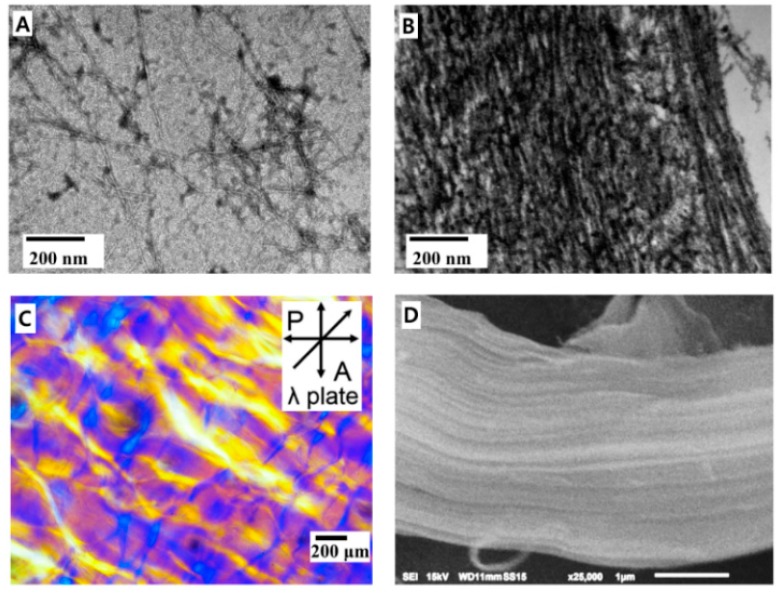
Morphological change of carboxylated cellulose nanofibers (CCNF) by conjugating catechol and silver nanoparticles. (**A**) TEM images of CCNF and (**B**) CCNF-DA/AgNPs; (**C**) polarized optical microscopy (POM) image of CCNF-DA with retardation (λ) plate; magenta and blue (or yellow) show disordered and anisotropic domains, respectively. The insect in (**C**) indicates the polarization directions of the polarizer (P) and analyzer (A); (**D**) SEM image of CCNF-DA.

Initially, CCNFs are randomly oriented in the solution (Figure 2A and Figure S1) and the composite films. After conjugation of DA to CCNF, anisotropically aligned CCNF domains were observed in CCNF-DA film by scanning electron microscopy (SEM) image (Figure 2D). Therefore, the nanostructure and hierarchical assembly of the CCNF composites were observed by TEM and POM (Figure 2C). POM suggests the orientation of CCNFs in the composite films. When the long axis of CCNF is designated as a direct vector (n), the POM image is dark when CCNFs in the film are disordered or n is parallel to either the polarizer (P) or analyzer (A). POM image with the retardation plate of 530 nm wavelength can show the detailed orientation. When the film is isotropic or disordered, a magenta color is developed. In contrast, blue/yellow domains show anisotropic ordering in the film when n is parallel or perpendicular, respectively, to the slow axis of the retardation plate. Specific morphology was not observed in the POM image of the CCNF film because the CCNFs are randomly oriented in the film as shown in the TEM data (Figure 2A). In contrast, the POM image of the CCNF-DA film shows lamellar domains with large size (~0.1 mm^2^) which are randomly distributed in the CCNF-DA film. This confirmed that CCNF-DAs are nematically aligned in each domain (Figure 2C). Unfortunately, the POM image of the CCNF-DA/AgNPs could not obtained due to the opacity of the film due to AgNPs (Figure S1).

In general, rigid nanofibers can only be self-assembled to nematic phase, *i.e.*, lamellar domain if there is a strong attraction between them. Initially, negatively charged CCNF in the film repel each other due to long ranged electrostatic repulsion and they were randomly ordered in the film. However, dopamine conjugation by replacing negatively charged carboxyl group of CCNF significantly reduces electrostatic repulsion between CCNFs and induces short-range attractions via hydrogen bonds, hydrophobic interaction and π–π stacking. These resulted in the nematic alignment of CCNF-DA in the film. In addition, oxidized catechol in the CCNF-DA is likely to induce additional covalent crosslinks between oxidized catechols, thereby stabilizing the anisotropic ordering. Therefore, we can observe anisotropic alignment in CCNF-DA phase in the POM images (Figure 2C).

In the case of the CCNF-DA/AgNPs film, catechol moiety in the CCNF-DA strongly binds to the silver to form both coordinative crosslinks between the silver and catechol, and the covalent crosslinks due to catechol oxidation. Aforementioned two crosslinks produced the anisotropic alignment of CCNF-DA/AgNPs in this film also (Figure 2B). Dopamine, Ag^+^ ions, and CCNF induced CCNF-DA/AgNPs a nanocomposite via strong interactions between these constituents to overcome other forces, eventually making an aligned stack structure. The overall structure of the CCNF-DA and the CCNF-DA/AgNPs can be approximated based on POM and TEM images as the typical structure of semi-crystalline polymers which consist of two phases: (i) an anisotropically aligned CCNF lamella phase and (ii) an amorphous inter-crystalline phase.

In nature, cellulose nanofibers without any surface modification form sophisticated 3-dimensional alignments [26,27] that contribute to their good mechanical properties [28]. The anisotropic alignment of fibrous materials has been emphasized to control mechanical properties in diverse practical applications including nanocomposites, tissue regeneration, stem cell therapy, and filters [29]. Although a single cellulose nanofiber has extraordinary stiffness (~130 GPa), the dried CCNF film does not have this property due to the inter-fibrillar slip by the electrostatic repulsive force. Therefore, self-assembly of CCNF via catechol chemistry can lead to the reinforcement of binding between nanofibers by overcoming the electrostatic repulsive force.

### 3.2. AgNPs in the CCNF Composite

**Figure 3:**
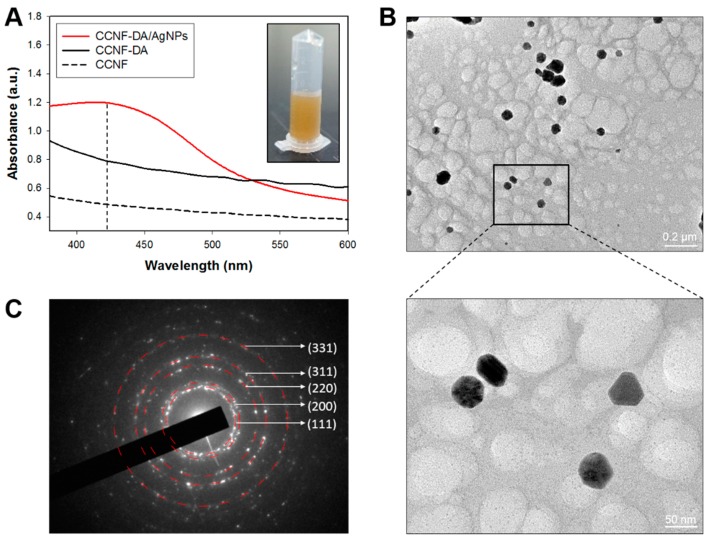
(**A**) UV–Vis spectroscopy of supernatant from CCNF, CCNF-DA and CCNF films. The insect in (**A**) shows the supernatant from the CCNF-DA/AgNPs film; (**B**) high-resolution transmission electron microscopy (HRTEM) image of extracted AgNPs. The inset black box in (**B**) indicates the area where the enlarged HRTEM image (bottom-right panel) was taken; (**C**) selected area electron diffraction (SAED) pattern of silver crystal of CCNF-DA/AgNP.

To characterize the intrinsic properties of the AgNPs, the AgNPs were separated from the CCNF-DA/AgNPs composite films via ultra-sonication in DW. This process gave a yellow heterogeneous solution, which showed a light absorption peak at around 400–440 nm in UV–Vis spectroscopy measurement (Figure 3A), probably due to the quantum plasmon resonance of AgNPs with diameters of ~10–90 nm [30]. The nano size of the AgNPs in the composite was also verified by the TEM image (Figure 3B and Figure S2A). The energy dispersive X-ray (EDX) spectra also confirmed that the resultant nanoparticles consisted primarily of Ag (Figure S2B). The selected area electron diffraction (SAED) pattern gave the characteristic peaks of crystalline Ag: (111), (200), (220), (311), and (331) planes (Figure 3C). These results suggest that the dopamine conjugates mediated the synthesis of crystalline AgNPs within the composite.

In order to synthesis such crystalline AgNPs, specific binding moieties for Ag^+^ ions are required to prevent aggregation and nucleate or growth [30]. Thus, we speculated that the catechol moieties of the CCNF-DA composites served as a specific Ag^+^ binder. Initially, the quadrupole in the DA could recruit Ag^+^ via cation-π interaction [31,32], and then the catechol moieties function as reducing agents for Ag^+^ ion because they have a lower standard electrode potential (*E*_o_ = 0.75 V) than silver (*E*_o_ = 0.799 V) [33]. Finally, the reduction of Ag^+^ due to catechol oxidation generates and stabilizes AgNPs in the composite.

### 3.3. Mechanical Tests

**Figure 4:**
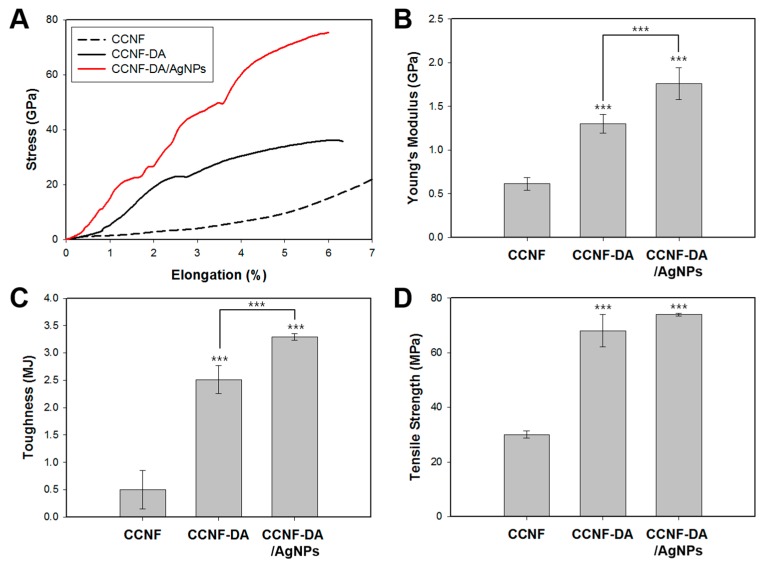
(**A**) Stress-strain curve; (**B**) tensile strength; (**C**) toughness; and (**D**) Young’s modulus of CCNF, CCNF-DA, and CCNF-DA/AgNPs films. The data of quadruplicate samples represent mean ± standard deviation with statistical significance (* *p* < 0.05, ** *p* < 0.01, *** *p* < 0.005; unpaired *t*-test).

To determine the contribution of the catechol moieties and the hierarchical structure of CCNF in catechol-AgNPs complexation, the mechanical properties of the CCNF, CCNF-DA and CCNF-DA/AgNPs films were evaluated. (Figure 4A). As expected, the covalent cross-links between the CCNF-DA as a result of the oxidation of catechol enhanced the tensile properties of the cast films. The stiffness (*E*_i_) and toughness of the CCNF-DA film were 1298 ± 107 MPa and 2.51 ± 0.26 MJ·m^−3^, respectively, which were approximately 2 and 0.5 fold higher than those of pure CCNF film (Figure 4B,C). This enhancement is presumably due to the crosslinking and the anisotropic alignment of CCNF due to the dopamine conjugation. It is well known that the quinones from dopamine oxidation can covalently couple to each other and this can mediate crosslinking between CCNFs [34]. Also, the anisotropic alignment of CCNF achieved by dopamine oxidation in CCNF contributed to the mechanical reinforcement of the CCNF-DA film. Furthermore, the addition of the coordinative complex between AgNPs and catechol in the CCNF-DA/AgNPs resulted in further increases in both stiffness (1759 ± 183 MPa) and toughness (3.29 ± 0.06 MJ·m^−3^). Many studies have proved that the catechol moiety in DA can form strong complexes with a variety of metal ions and metal oxides [11,35]. This complexation between the catechol and silver enhanced the mechanical properties of the CCNF-DA/AgNPs film.

### 3.4. Conductivity Test

ICP analysis on the CCNF-DA/AgNPs film suggests that the weight % of AgNPs in the film is about 6.79. The AgNPs in a film generally make the composite conductive. Therefore, the electrical conductivity of the CCNF-DA/AgNPs film surface measured using 4-probe method was ~4 S·cm^−1^, which is higher than other conductive nanocellulose composite films in the considering with the conducting material content (Table 1) [36,37,38,39]. The reason would be the difference in the Ag reduction method. Dipping process in AgNO_3_ solution leads to interface-dominant AgNPs deposition on the composite film, giving higher electrical conductivity. However, it should be noted that the AgNPs deposition possibly occurred inside the composite film as the catechol oxidation-mediated CCNF alignment is observed in the cross-section of the composite film (Figure 2B). The electrical conductivity was compatible to ~0.16–10 S·cm^−1^ of graphene-based wearable and flexible devices [40,41]. Thus, the CCNF-DA/AgNPs composite system has the potential to be used as a substrate material for wearable flexible electronics. Electrically conductive materials are recognized as promising scaffolds to regenerate neurons or nerve tissues for therapeutic purposes by electric stimuli. The electrical conductivity of the CCNF-DA/AgNPs film is high enough to develop a scaffold for nerve tissue regeneration [42].

### 3.5. Antibacterial Test

**Figure 5:**
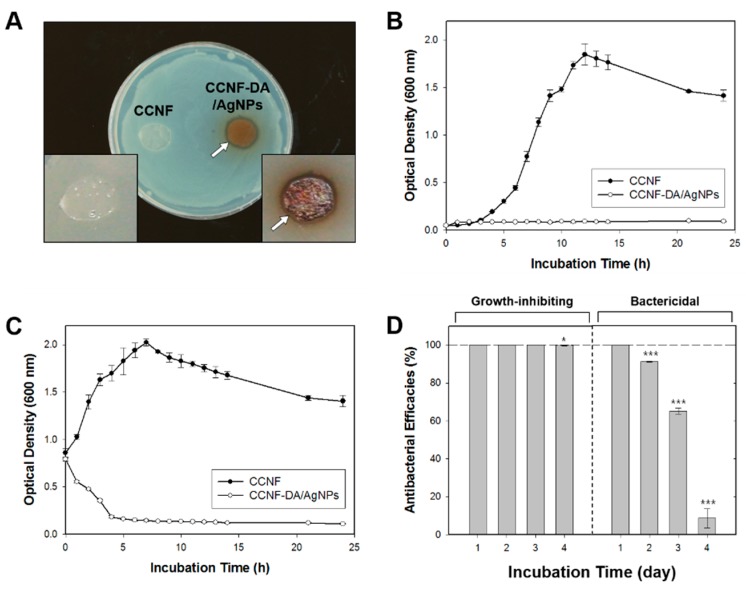
Antibacterial test on CCNF-DA/AgNPs membrane. (**A**) Disk diffusion test; (**B**) bacterial growth profiles; and (**C**) bactericidal profiles of the CCNF-DA/AgNPs membrane against *E. coli*; (**D**) Growth-inhibiting (**left**) and bactericidal (**right**) efficacies of CCNF-DA/AgNPs membrane for a long period. White arrows indicate the inhibition zone. The data represent mean ± standard deviation with statistical significance (* *p* < 0.05, ** *p* < 0.01, *** *p* < 0.005; unpaired *t*-test).

The antibacterial activity of the CCNF-DA/AgNPs film was evaluated using the disk diffusion test against Gram-negative (*E. coli*) and one Gram-positive (*S. aureus*) bacteria (Figure 5A and Figure S3A). The CCNF film was used as a negative control. The CCNF-DA/AgNPs film exhibited effective antibacterial activity, showing significant inhibition zones with the diameter of 718.45 ± 87.17 mm^2^ toward *E. coli* and 1056.57 ± 140.18 mm^2^ toward *S. aureus*, whereas there was not any inhibition of bacterial growth on the CCNF film. This clearly demonstrates that the antibacterial activity is not due to the CCNF film but only due to the AgNPs impregnated on the CCNF-DA/AgNPs film.

In addition, the sample films were incubated in liquid LB media with log-phase of *E. coli* and *S. aureus*. Subsequently, we performed quantitative time profiling of growth-inhibiting activity for 1 day (Figure 5B and Figure S3B). Similar to the results of the disk diffusion test, the CCNF-DA/AgNPs film inhibited the growth of both tested bacterial strains effectively over the course of 24 h. Further, the CCNF-DA/AgNPs film showed bactericidal effects against both bacterial strains (Figure 5C and Figure S3C). Notably, most of these bacteria were killed on the CCNF-DA/AgNPs film within 5 h. The CCNF film did not show any growth-inhibiting and bactericidal activity against bacterial strains. We concluded that the AgNPs impregnated on the CCNF-DA/AgNPs film have good antibacterial activity against both Gram-negative and Gram-positive bacteria.

To evaluate the long-term antibacterial ability of the CCNF-DA/AgNPs film, growth-inhibiting and bactericidal efficacies were measured for a period of 5 days (Figure 5D). The growth-inhibiting efficacy was perfectly maintained during the first 3 days and decreased gradually afterward. In addition, we found that the bactericidal efficacy remained ~100% for the first 2 days but was completely lost after 5 days. After the 5th day, the CCNF-DA/AgNPs film had become limp and shapeless due to several washing processes and prolonged exposure to the wet environment. The decrease of growth-inhibiting and bactericidal abilities might be caused by the AgNPs release to certain extent in medium. The decrease of growth-inhibiting and bactericidal abilities might be caused by the AgNPs release to certain extent in medium. After the CCNF-DA/AgNPs composite was incubated in PBS buffer at 37 °C for 2 days, the silver weight % of the composite film was decreased to 3.83 as determined by ICP. Thus, AgNPs are possibly released in LB medium because catechol moieties are gradually oxidized to o-benzoquinone that has week adhesion in wet conditions [43]. Furthermore, the bactericidal activity assay employed harsh conditions of high bacterial cell density (1 × 10^9^ cells·mL^−1^). Considering its effective growth-inhibiting and bactericidal abilities, we expect that the antibacterial activity of the CCNF-DA/AgNPs film could be sustainable for a longer period in practical applications such as wound dressing and water filter.

## 4. Conclusions

In this paper, a mussel-inspired composite of cellulose nanofiber (CCNF) and silver nanoparticles (AgNPs) with improved mechanical properties, antimicrobial activity, and electrical conductivity were developed by conjugating the catechol moieties on CCNF and *in situ* silver reduction. The catechol conjugation and the addition of AgNPs induced anisotropic self-alignment of the cellulose nanofibers which enhances electrical and mechanical properties of the composite. The composite showed a surface electrical conductivity of 4 S·cm^−1^ and excellent antibacterial efficacy against both Gram-positive and Gram-negative bacteria. Our AgNP/catechol/cellulose nanofiber composite system holds great promise as a material for the biomedical devices and wearable electronics.

## Figures and Tables

**Table 1:** Conductivity of CCNF, CCNF-DA and CCNF-DA/AgNPs.

Sample	Conductivity (S·cm^−1^)
CCNF	<10^−6^
CCNF-DA	<10^−6^
CCNF-DA/AgNPs	~4
Cellulose nanofiber/polypyrrole/AgNPs [36]	3 × 10^−3^
Cellulose/polypyrrole aerogel [37]	0.08
Bacterial cellulose/polypyrrole [38]	77
Polyurethane/gold nanoparticle [39]	1–1,000

## Supplementary Materials

The following are available online at www.mdpi.com/2073-4360/8/3/102/s1. Figure S1: POM image of CCNF and CCNF-DA/AgNPs hydrogels; Figure S2: Size distribution of AgNPs on CCNF-DA/AgNPs and EDS analysis of the elemental composition of CCNF-DA/AgNPs, Figure S3: Antibacterial test on CCNF-DA/AgNPs membrane against *S. aureus*.
