# Supplementary material for: Mussel-Inspired Anisotropic Nanocellulose and Silver Nanoparticle Composite with Improved Mechanical Properties, Electrical Conductivity and Antibacterial Activity
Source: Polymers (Basel). 2016 Mar 22;8(3):102. doi: 10.3390/polym8030102 (PMC6432548; doi:10.3390/polym8030102)
Supplement: Supplementary file 1 [file polymers-08-00102-s001.pdf]

Supplementary Materials: Mussel-Inspired Anisotropic Nanocellulose and Silver Nanoparticle Composite with Improved Mechanical Properties, Electrical Conductivity and Antibacterial Activity

Hoang-Linh Nguyen, Yun Kee Jo, Minkyu Cha, Yun Jeong Cha, Dong Ki Yoon, Naresh D. Sanandiya, Ekavianty Prajatelistia, Dongyeop X Oh and Dong Soo Hwang

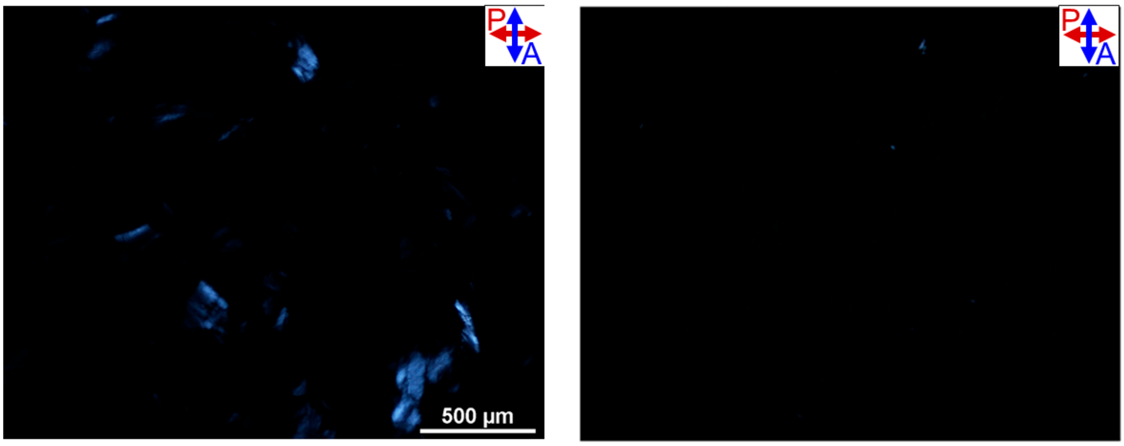

(**A**) (**B**)

Figure S1. POM images of (**A**) CCNF and (**B**) CCNF-DA/AgNPs hydrogels. Both images are scaled to the same magnification.

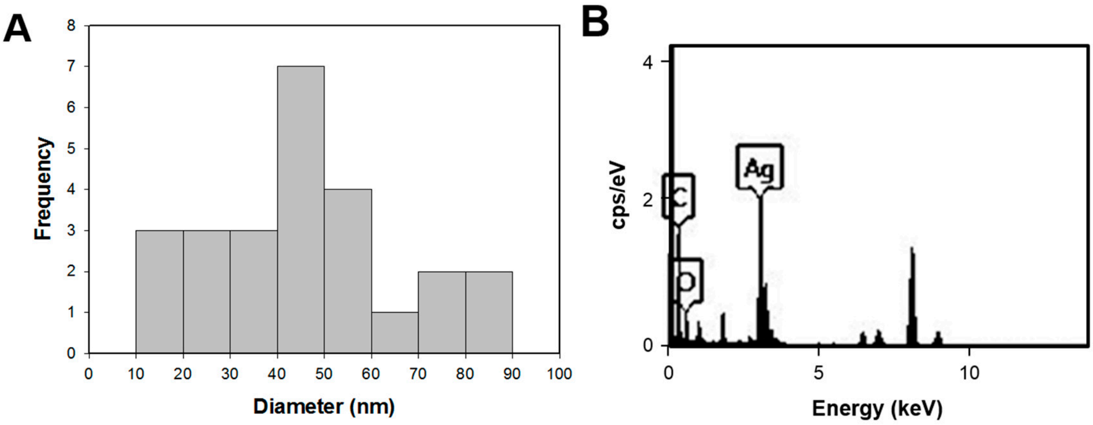

Figure S2. (**A**) Size distribution of AgNPs on CCNF-DA/AgNPs; (**B**) EDS analysis of the elemental composition of CCNF-DA/AgNPs.

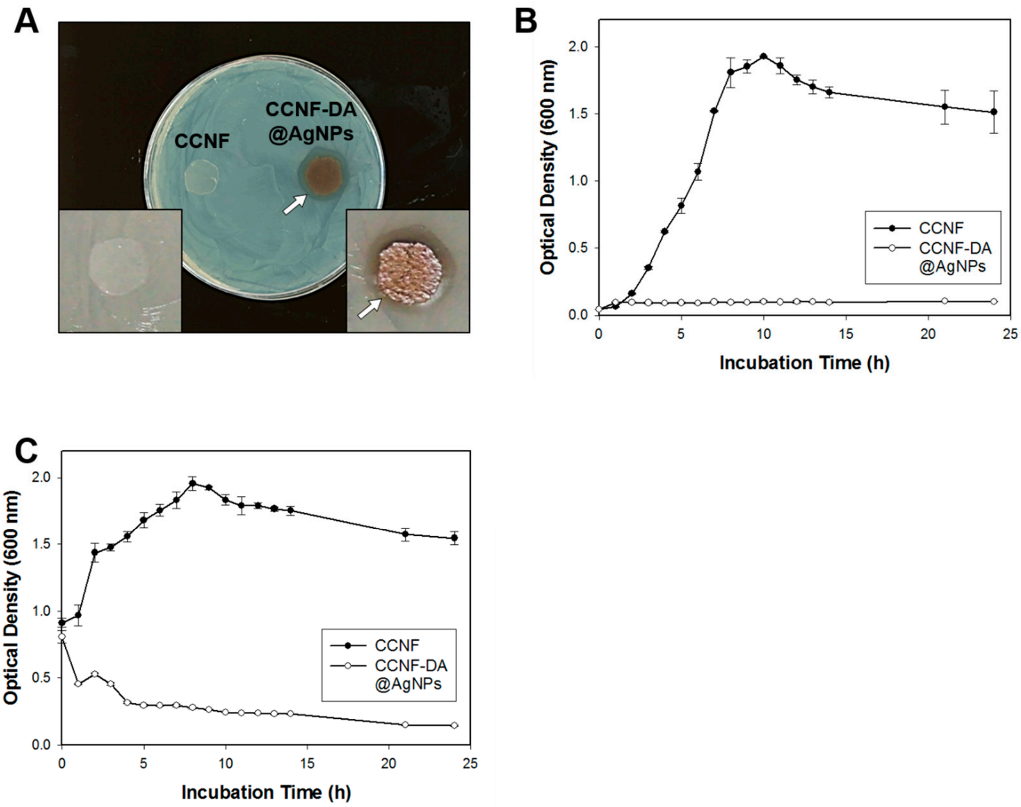

Figure S3. Antibacterial test on CCNF-DA/AgNPs membrane. (**A**) Disk diffusion test; (**B**) bacterial growth profiles; and (**C**) bactericidal profiles of the CCNF-DA/AgNPs membrane against *S. aureus*. The data represent mean ± standard deviation. The white arrow indicates the inhibition zone.

© 2016 by the authors; licensee MDPI, Basel, Switzerland. This article is an open access article distributed under the terms and conditions of the Creative Commons by Attribution (CC-BY) license (http://creativecommons.org/licenses/by/4.0/).
